# Isolation and molecular characterization of the *Salmonella* Typhimurium orphan phage Arash

**DOI:** 10.1186/s12866-023-03056-9

**Published:** 2023-10-19

**Authors:** Mohammad Hashem Yousefi, Jeroen Wagemans, Seyed Shahram Shekarforoush, Marta Vallino, Nadiia Pozhydaieva, Katharina Höfer, Rob Lavigne, Saeid Hosseinzadeh

**Affiliations:** 1 Department of Food Hygiene and Public Health, School of Veterinary Medicine, Shiraz University, Shiraz, 71946- 84471 Iran; 2 Department of Biosystems, KU Leuven, Leuven, 3001 Belgium; 3 Institute of Sustainable Plant Protection, National Research Council of Italy, Turin, 10135 Italy; 4 Max Planck Institute for Terrestrial Microbiology, SYNMIKRO, Karl-von-Frisch-Strasse 16, Marburg, 35043 Germany

**Keywords:** Bacteriophage, *Salmonella* Typhimurium, Novel phage genus, ‘Arashvirus’

## Abstract

The current threat of multidrug resistant strains necessitates development of alternatives to antibiotics such as bacteriophages. This study describes the isolation and characterization of a novel *Salmonella* Typhimurium phage ‘Arash’ from hospital wastewater in Leuven, Belgium. Arash has a myovirus morphology with a 95 nm capsid and a 140 nm tail. The host range of Arash is restricted to its isolation host. Approximately 86% of the phage particles are adsorbed to a host cell within 10 min. Arash has latent period of 65 min and burst size of 425 PFU/cell. Arash has a dsDNA genome of 180,819 bp with GC content of 53.02% with no similarities to any characterized phages, suggesting Arash as a novel species in the novel ‘Arashvirus’ genus. Arash carries no apparent lysogeny-, antibiotic resistance- nor virulence-related genes. Proteome analysis revealed 116 proteins as part of the mature phage particles of which 27 could be assigned a function. Therefore, the present findings shed light on the morphological, microbiological and genomic characteristics of Arash and suggest its potential application as therapeutic and/or biocontrol agent.

**Supplementary Information:**

The online version contains supplementary material available at 10.1186/s12866-023-03056-9.

## Introduction

Non-typhoidal *Salmonella* spp. (NTS) are considered as one of the most serious zoonotic foodborne pathogens causing 155,000 deaths from diarrhea annually worldwide [1, 2]. It is the most frequently isolated pathogens in foodborne outbreaks in the European Union, with more than 91,000 salmonellosis cases each year [3, 4]. In Belgium alone, approximately 3,000 people become infected annually [5]. The symptoms of salmonellosis include fever, nausea, vomiting, abdominal cramps and inflammatory diarrhea. Furthermore, salmonellosis can lead to bacteremia and septicemia, followed by hospitalization and even death in severe cases, particularly in immunodeficient persons [6]. Around 50% of NTS infections are estimated to originate from contaminated food products [7, 8] with fresh-cut products, raw and under-cooked meat, poultry and eggs being highly reported food commodities with *Salmonella* contamination [9, 10].

Among the NTS serovars, *S.* Typhimurium and *S.* Enteritidis are the most important serovars that cause self-limiting enterocolitis in humans [11]. Especially *S.* Typhimurium is more critical because of its broad host range and zoonotic potential [12]. Moreover, multidrug resistant (MDR) *Salmonella* strains of both serovars have been isolated from humans, animals and the human food chain [13, 14]. MDR *Salmonella* strains are resistant to clinically relevant antibiotics including third-generation cephalosporins and fluoroquinolones [15, 16]. The emergence of these MDR strains results in economic losses in food production industries and complicates their containment [17–19]. In addition, MDR strains are responsible for the majority of *S.* Typhimurium outbreaks [20]. Inappropriate use of antibiotics in poultry and other farm animals is one of the reasons for the urging problem with MDR *Salmonella* strains in clinical samples [21].

Phages are types of viruses that specifically infect bacterial cells as their host and replicate only in them and are almost 50 times smaller than their host cells [22]. They are ubiquitous in environmental sources including human and animal feces, sewage and food [23]. Today, bacteriophages are receiving attention as antibacterials to deal with antibiotic-resistant pathogens in medicine, veterinary medicine, and food and agriculture industries. This is explained by their high specificity (up to the strain level), self-replication and rapid antimicrobial action. They are also low-cost to produce and can readily be isolated from all natural environments [24]. Phages do not harm eukaryotic cells and have received a “Generally Recognized As Safe” status. They also received halal and kosher certifications [25–28]. Some commercial phage products like SalmoFresh, Armament and Salmonelex have been marketed for biocontrol of *Salmonella* spp. in food products [24, 29]. Moreover, phages have played a very key role in understanding the fundamental principles of molecular biology. The identification of CRISPR-Cas anti-phage defense systems followed by the development of the concept of gene editing has led to enormous developments in biology [30, 31]. Phages also can be employed in advanced biotechnological applications including bacterial detection, drug delivery vehicles, vaccine development and designing cheap and stable sensors for diagnostic assays [32].

In the present study, we isolated and characterized a novel *S.* Typhimurium bacteriophage from hospital and municipal sewage from Belgium. Based on whole genome sequencing, phage Arash represents a new genus with therapeutic or biocontrol potential in further applications.

## Materials and methods

### Phage isolation, purification, and propagation

Phage isolation was based on the method previously presented by Wang et al. (2017) with slight modifications [33]. Several hospital and municipal sewage samples were collected aseptically and kept at 4 °C for 24 h to allow settling of the debris and bigger particles. Next, 10 ml of each sample were centrifuged at 6,000 ×g for 10 min at 4 °C, followed by filtration of the supernatant through 0.22 μm syringe filters. 100 µl of each filtrate and 300 µl of an overnight culture of *S.* Typhimurium ATCC 14,028 were added to 50 ml of Lysogeny broth (LB) medium and incubated for 24 h at 37 °C with agitation (160 rpm). After incubation, the suspension was centrifuged for 10 min at 4 °C and 6,000 ×g and filtered again. The filtrate was then serially diluted in phage buffer (10 mM Tris.HCl, 150 mM NaCl, 10 mM MgSO_4_; pH 7.5). Subsequently, 100 µl of each dilution was mixed with 300 µl of an overnight bacterial suspension in 4 ml of soft agar (LB broth with 0.7% [w/v] agar) and poured onto solid LB agar plates (1.5% [w/v] agar) (double layer agar (DLA) method). Once the top layer was completely set, plates were incubated overnight for 18 h at 37 °C. Observation of transparent plaques indicated lack of bacterial growth. A single plaque was picked and suspended in 3 ml phage buffer, diluted and re-cultured using the DLA method. To ensure purity of the phage, this stage was repeated three times.

To propagate the isolated phage for further use and storage first, 10 ml of a fresh bacterial suspension with an OD at 600 nm (OD_600_) of 0.6 (approximately 10^8^ CFU/ml) was added to 100 ml of LB supplemented with 10 mM MgSO_4_, and the mixture was incubated at 37 °C. After 1 h, 100 µl of phage suspension was added and the incubation continued at 37 °C and 160 rpm for 24 h. Then, the mixture was centrifuged at 6,000 ×g for 10 min, the supernatant was filtered, and the phage titer was counted for further uses.

### Morphology analysis by Transmission Electron Microscopy

The method presented by Vallino et al. (2021) was used for taking transmission electron micrographs (TEM) [34]. Briefly, 10 µl of the pure phage stock was deposited on carbon and formvar- coated 400 mesh grids (Gilder, Grantham Lincolnshire, England) and negatively stained with aqueous 0.5% w/v uranyl acetate. Observations and photographs were made using a Philips CM 10 transmission electron microscope (Eindhoven, The Netherlands), operating at 60 kV.

### Phage titration and host range testing

To determine the phage titer and bacterial susceptibility, 100 µl of an overnight bacterial culture was plated using the DLA method. Once the top layer was solidified, diluted phage suspensions were dropped on the top layer in equal amounts of 10 µl. The plates were incubated at 37 °C for 24 h. For the host range assay, several Gram-negative bacterial strains, including *Salmonella* spp., *Hafnia alvei, Morganella morganii, Citrobacter* spp., *Escherichia coli* and *Klebsiella* spp. were tested for phage susceptibility (Table 1). This was performed in triplicate. The *Salmonella* strains were serotyped using whole genome sequencing (Illumina MiniSeq, 2*150 bp, Nextera Flex library kit), followed by a SeqSero2 v1.1.0 analysis [35].

### Phage adsorption assay

Bacteriophage adsorption was determined by enumeration of non-adsorbed phage to the host (*S.* Typhimurium ATCC 14,028) bacteria (free phage). For this purpose, the method described by Denes et al., 2015 was applied, with some modifications [36]. First, a phage suspension at 10^7^ PFU/ml and a fresh exponential bacterial culture (OD_600_ of 0.6) was prepared. One hundred µl of the phage suspension was added to 1 ml of the bacterial culture to have a multiplicity of infection (MOI) of 0.01. The infected cultures were agitated. Next, 5, 10 and 15 min after the start of infection, 100 µl aliquots were taken, quickly diluted with 900 µl of phage buffer and passed through a 0.22 μm filter to obtain the free phage. Finally, the free phage count was determined in triplicate. The phage count at time 0 was measured by adding the phage suspension to a sterile culture (without host). The adsorption curve was measured three times.

### One-step growth curve

The one-step growth curve of the phage was determined as described by Duc et al. (2018), with some modifications [37]. The phage suspension (MOI of 0.01) was mixed with 10 ml of *S.* Typhimurium ATCC 14,028 culture (OD_600_ of 0.3 equivalent to 10^8^ CFU/ml). The mixture was incubated for 15 min at 37 °C to allow adsorption. Subsequently, the suspension was diluted (10^− 3^, 10^− 4^, 10^− 5^; 10^− 6^) and incubated at 37 °C for 180 min. An aliquot was taken every 5 to 10 min and titered. The experiment was performed in three replicates. The burst size was determined by dividing the number of phages formed during the rise period with the estimated number of infected cells at the latent period time [38].

### Killing curve

Killing curves were performed according to James et al. (2020) [39]. Fresh bacterial culture (*S.* Typhimurium ATCC 14,028) at an OD_600_ of 0.6 (∼10^8^ CFU/ml) (total volume of 200 µl in a 96-well microplate) was infected with different concentrations of phage to obtain final MOIs of 0.1, 1, 10 and 100. Positive and negative controls were also considered by excluding the phage or bacterial cells, respectively. Finally, the plate was incubated at 37 °C in a microplate reader (CLARIO star Plus, BMG Labtech, Ortenberg, Germany) for 13 h. The OD_600_ of each well was measured at 30 min intervals. The experiment was performed in three replicates.

### Whole genome sequencing & proteome analysis

Phage DNA isolation, whole genome sequencing, and annotation was performed as described in Azari et al. (2023) [40]. Briefly, Sequencing was carried out on an Illumina (San Diego, CA, USA) MiniSeq device. The raw sequencing data were assembled using SPAdes [41] and then the most related phages were identified using BLASTn [42] and Viptree v1.9 [43]. To investigate phage taxonomy, intergenomic similarities was calculated using VIRIDIC [44] and vConTACT2 [45]. Genome annotation was executed with RASTtk [46], BLASTp and HHPred [47]. A genome was created with Easyfig [48].

A phage suspension with a concentration of approximately 10^10^ PFU/ml was used to determine the phage’s structural proteome. First, Arash was purified using a sucrose gradient ranging from 0 to 45%. The gradient was created in TM buffer (50 mM Tris-HCl, 10 mM MgCl_2_, pH 7.5). 500 µL of the phage solution was layered onto the top of the gradient. Subsequently, centrifugation was performed at 70,000 x g, 20 min, 4 °C. The resulting gradient fraction, containing the phages, was collected using a blunt cannula and then transferred to a new ultracentrifugation tube. To the collected fraction, 30 mL of ice-cold TM buffer were added. The phage particles were then pelleted through centrifugation at 100,000 x g, 1 h, 4 °C. After discarding the supernatant, the pellet was resuspended in 500 µL of TM buffer and incubated at 4 °C overnight. The isolated phages were subjected to lysis buffer (50 mM Tris-HCl, pH 7.5, 1% SLS, 2 mM TCEP) and heated to 95 °C, 10 min. A sonication step (10 s, 20% amplitude, 0.5 pulse) was performed to degrade nucleic acids in the samples. Following this, iodoacetamide was added to the final concentration of 4 mM, and the samples were incubated for 30 min under light protection. The proteins were precipitated using acetone. The resulting pellets were washed with 500 µL of methanol (-80 °C), air-dried, and resuspended in 50 µL of resuspension buffer (50 mM Tris-HCl, pH 7.5, 0.5% SLS). The protein concentration was determined using the BCA assay (Pierce TM, BCA protein assay kit, ThermoFisher Scientific, Waltham, MA, USA). To 10 µg of isolated proteins, 0.5 µg of sequencing-grade trypsin (Promega) was added. Digestion was carried out overnight at 30 °C. The remaining SLS was precipitated by adding 1.5% TFA (v/v) and subsequent centrifugation at 4 °C, 17,000 × g, 10 min. The resulting supernatant was desalted for mass spectrometric analysis using C18 solid-phase columns (Chromabond C18 spin columns, Macherey Nagel, Düren, Germany) and then analyzed using liquid-chromatography-mass spectrometry (LC-MS) carried out on an Exploris 480 instrument connected to an Ultimate 3000 RSLC nano and a nanospray flex ion source (all Thermo Scientific). Peptide separation was performed on a reverse phase HPLC column (75 μm x 42 cm) packed in-house with C18 resin (2.4 μm; Dr. Maisch). The following separating gradient was used: 98% solvent A (0.15% formic acid) and 5% solvent B (99.85% acetonitrile, 0.15% formic acid) to 30% solvent B over 45 min at a flow rate of 300 nl/min.

The data acquisition mode was set to obtain one high resolution MS scan at a resolution of 60,000 full width at half maximum (at m/z 200) followed by MS/MS scans of the most intense ions within 1 s (cycle 1s). To increase the efficiency of MS/MS attempts, the charged state screening modus was enabled to exclude unassigned and singly charged ions. The dynamic exclusion duration was set to 14 s. The ion accumulation time was set to 50 ms (MS) and 50 ms at 17,500 resolution (MS/MS). The automatic gain control (AGC) was set to 3 × 106 for MS survey scan and 2 × 105 for MS/MS scans.

For spectral based assessment MS raw files searches were carried out using MSFragger embedded within Scaffold 4 (Proteome Software) with 20 ppm peptide and fragment tolerance with Carbamidomethylation (C) as fixed, and oxidation (M) as variable modification using a customized phage protein database (based on the phage genome annotation).

### NCBI accession number

The genome information is available on NCBI GenBank under accession number OQ632216.

## Results and discussion

Among raw samples from various sources, four phages were isolated: two from hospital sewage, one form municipal sewage and the last one from a pig farm. Since three phages were very similar to previously described *Chivirus* phages, this study will focus on one novel phage isolated from hospital sewage (University Hospitals Leuven), which was called Arash. The naming of this phage was inspired by the Persian mythology heroic figure, Arash the Archer (Āraš-e Kamāngīr).

### Arash is a myovirus with a narrow host range

**Fig. 1:**
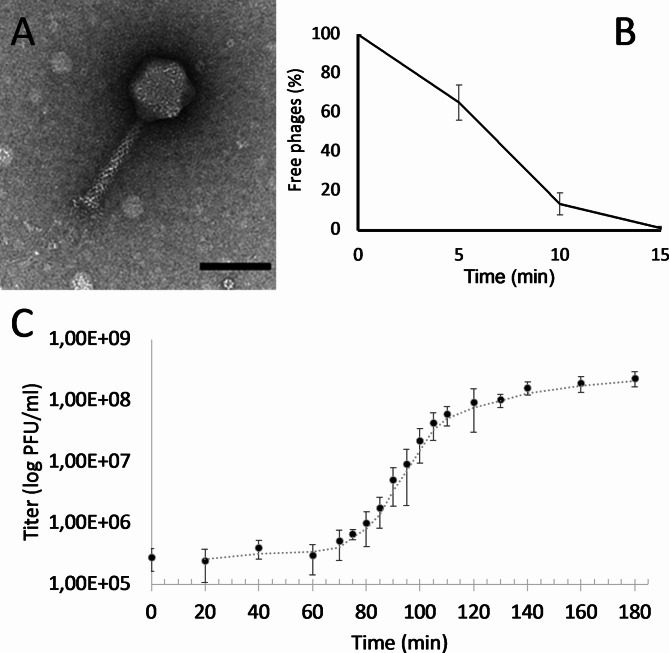
Microbiological characterization of phage Arash. (**A**) Electron micrograph: TEM analysis shows a myovirus morphology for Arash (scale bar represents 100 nm). (**B**) Adsorption curve of Arash: more than 86% of the phages are being bound in the first 10 min followed by a gradual increase to 98.8% in the next 5 min. (**C**) One-step growth curve: Arash has a latent period of 65 min, with a high burst size of 425 PFU/cell

TEM revealed that Arash is a myovirus with an icosahedral head (95.80 ± 2.04 nm in diameter) and a contractile tail (140.47 ± 1.05 nm in length) (Fig. 1A). Its host range data is presented in Table 1. Arash forms a lysis zone for seven bacterial strains out of 19 tested strains, including a reference strain (the isolation host), several *Salmonella* Enteritidis strains, two *H. alvei* strains isolated from retail chicken and one *E. coli* strain isolated from milk. However, it only produces plaques on its isolation host strain *Salmonella* Typhimurium ATCC 14,028 (Supplementary Figure S1) and therefore can be considered a narrow host range spectrum based on the available collection of strains.

**Table 1:** Host range analysis of phage Arash

Species	Strain	Source	Lysis*
*Salmonella* Typhimurium	ATCC 14,028	Reference strain	+
*Salmonella* Typhimurium	LT2	Reference strain	-
*Salmonella* Enteritidis	ATCC 13,076	Reference strain	LFW
*Salmonella* Enteritidis	ATCC 13,046	Reference strain	LFW
*Salmonella* Enteritidis	S42	Retail chicken	LFW
*Salmonella* Enteritidis	S2	Retail chicken	-
*Klebsiella pneumoniae*	2	Human infection	-
*Klebsiella* spp.	Klb	Wild-unknown source	-
*Hafnia alvei*	S44	Retail chicken	LFW
*H. alvei*	S31	Retail chicken	-
*H. alvei*	S439	Retail chicken	LFW
*Citrobacter* spp.	S53	Retail chicken	-
*Morganella morganii*	S257	Retail chicken	-
*M. morganii*	S28.1	Retail chicken	-
*E. coli*	MG1655	K12 reference strain	-
*E. coli*	E95	Milk	LFW
*E. coli*	E94	Milk	-
*E. coli*	E96	Milk	-
*E. coli*	E105	Milk	-

*Productive infection: +; Lysis from without: LFW; no lysis: -

### Microbiological characterization of Arash

**Fig. 2:**
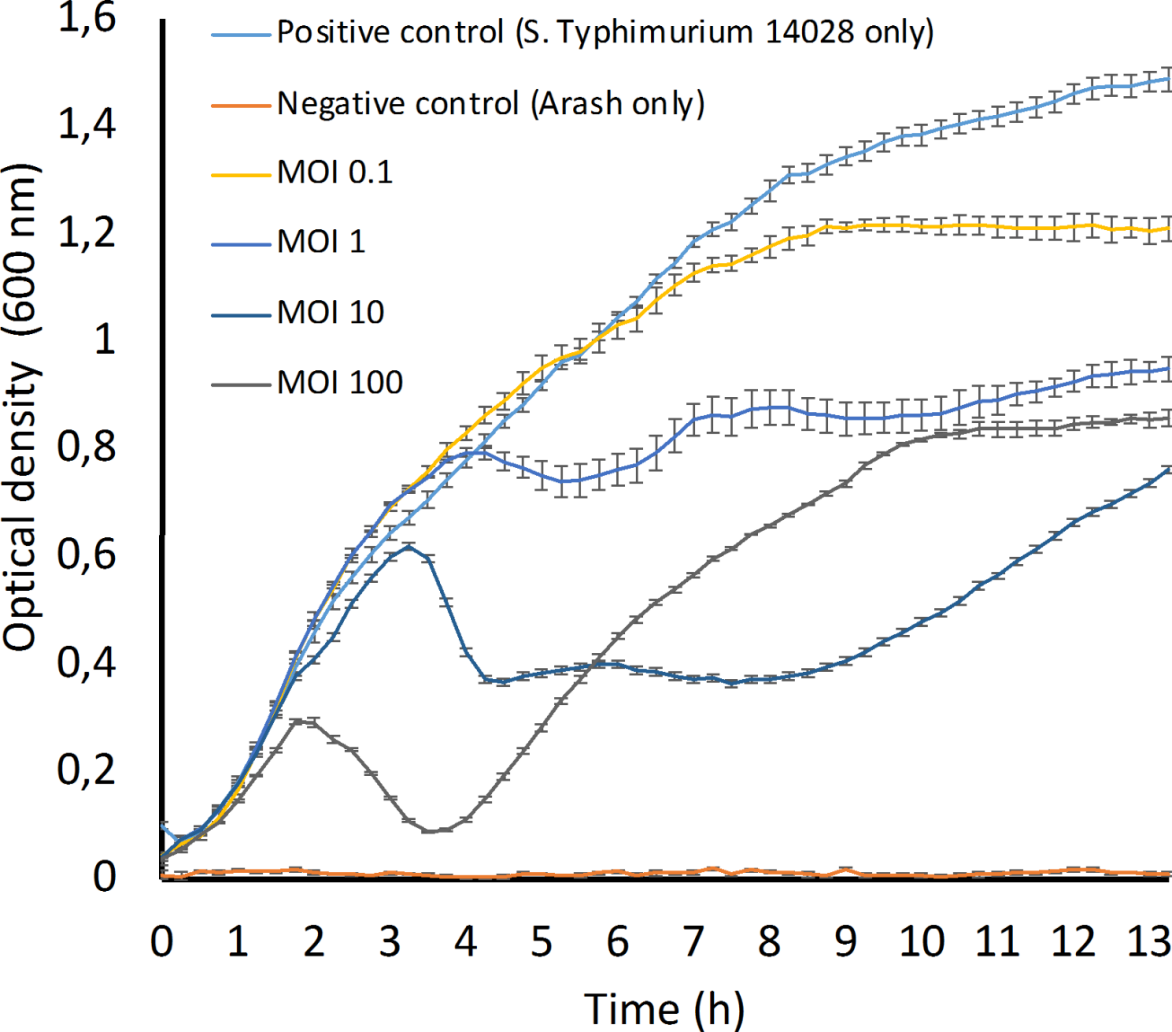
Killing curve of phage Arash in different MOIs. *S.* Typhimurium 14,028 was infected with Arash at different MOIs. The optical density was followed over time. Each point represents the average of three replicates and its standard deviation. As positive control, a culture without phage was monitored. As negative control, a phage only sample was measured over time

Arash adsorbs relatively efficiently, with more than 86% of the phages being bound in the first 10 min followed by a gradual increase to 98.8% in the next 5 min (Fig. 1B). As explained by Abedon (2011), one key factor in measuring the antibacterial effect of a phage is phage adsorption. Therefore, phages like Arash which have a relatively fast adsorption rate are more likely to be beneficial for biocontrol or therapy purposes [49]. A one-step growth curve (Fig. 1C) indicates that Arash has a latent period of 65 min, with a relatively high burst size of 425 PFU/cell, also beneficial for future applications. Next, killing curves were made. As illustrated in Fig. 2, the higher the MOI, the faster the bactericidal activity is observed. At an MOI of 100, regrowth of a resistant population is observed around 3 to 4 h after infection. At an MOI of 10, the population seems to be controlled for the longest period. According to Islam et al. (2020a), phage LPST153 at MOIs of 0.1, 1, 10 and 100 was able to inhibit the growth of *S.* Typhimurium ATCC 13,311 in 12 h [50]. In another study by Islam et al. (2020b), the growth of *S.* Typhimurium UK-1 was efficiently inhibited by LPST94 at MOI 1 over 12 h. This phage also inhibited the growth of *S.* Typhimurium UK-1, *S.* Typhimurium ATCC 14,028, *S.* Enteritidis ATCC 13,076 and *S.* Enteritidis SGSC 4901 for 11–12 h at MOIs of 0.1, 1, 10 and 100 [51]. According to Esmael et al. (2021), the growth of *S.* Typhimurium EG.SmT3 was inhibited by the phages SPHG1 and SPHG3 for 6 h at 0.1, 1, and 5. However, the application of MOI 0.01 did not successfully inhibit the growth of bacteria as much as the other MOIs, especially after 2 h [52].

### Arash is a novel phage from a yet unclassified genus

**Fig. 3:**
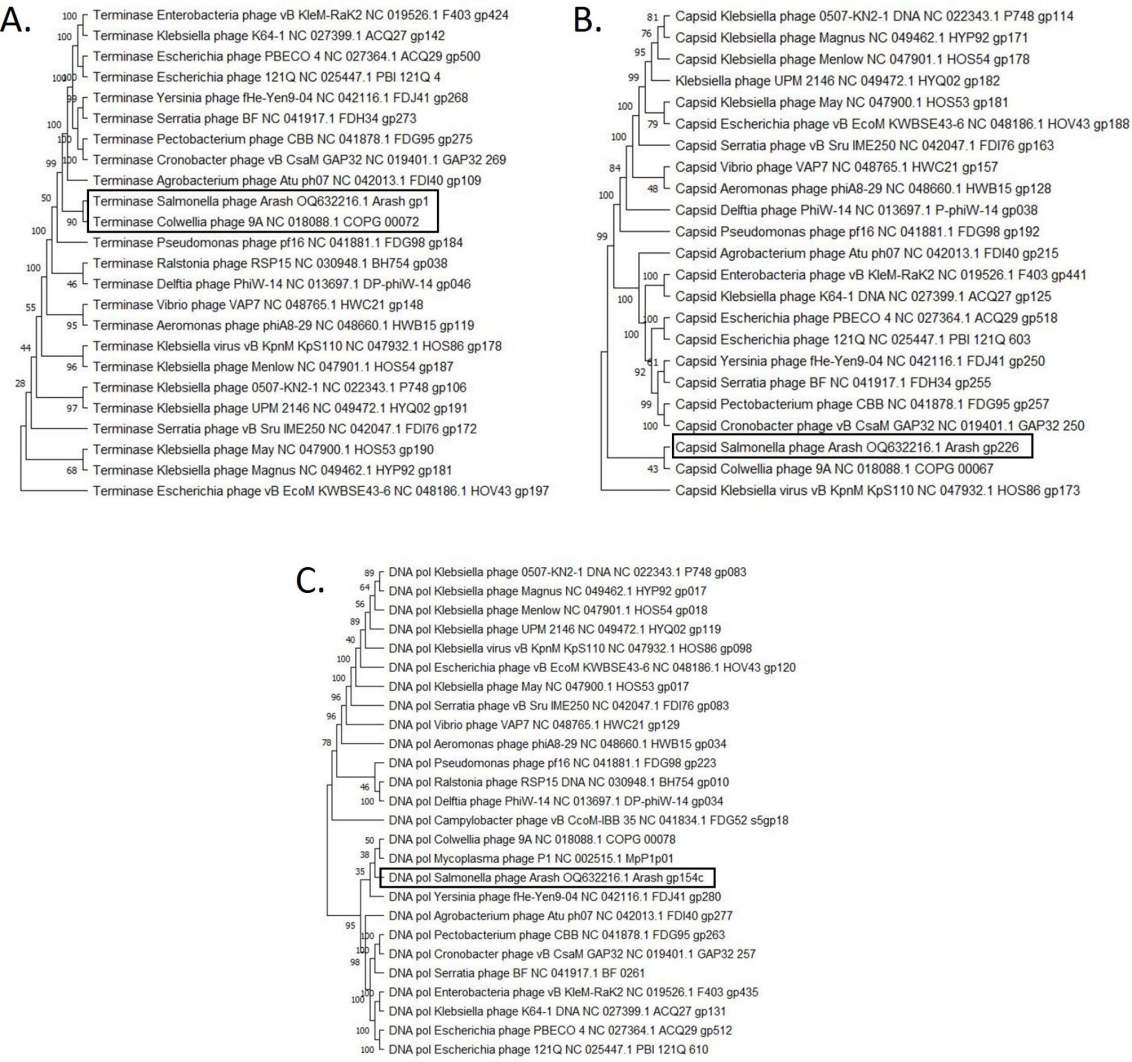
Phylogenetic trees of Arash terminase (large subunit) (**A**), major capsid protein (**B**) and DNA polymerase (**C**). The consensus trees were constructed using the corresponding protein of Arash and related phages with MEGA X [53]. The amino acid sequences were aligned using MUSCLE and a neighbour-joining tree with 1,000 bootstraps was constructed

Whole genome analysis revealed that phage Arash has a dsDNA genome of 180,819 bp with a GC content of 53.02%. Both BLASTn and Viptree showed no significant similarities to any previously characterized phages. Therefore, phage Arash can be considered a novel species in the novel ‘Arashvirus’ genus within a yet unclassified family (Supplementary Figure S2). This was confirmed using a vConTACT2 gene-sharing network analysis, identifying Arash as a singleton when compared to the NCBI viral RefSeq database. Next, a neigbour joining-tree (1,000 bootstraps) was constructed for the terminase protein, the major capsid protein and the DNA polymerase to compare these Arash proteins to the corresponding proteins of phages identified by VipTree as having a similar proteome (Fig. 3). Only the terminase of Arash clusters consistently (in 90% of the bootstraps) together with a known terminase of *Colwellia* phage 9A (NC_018088.1). However, on the nucleotide level, there is no similarity at all between Arash and 9A.

**Fig. 4:**
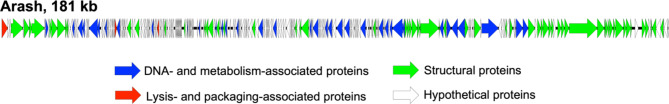
Genome map of Arash. Each arrow represents a coding sequence. In red, genes encoding packaging and lysis-associated proteins are displayed, in green structural proteins and in blue DNA- and metabolism-associated proteins (adapted from EasyFig)

Based on the structural annotation, 252 coding sequences and 21 tRNAs were identified (Supplementary Table S1). Only 50 coding sequences encode proteins that could be assigned a putative function (19.8%), while the remaining CDSs (80.2%) encode hypothetical proteins (Fig. 4). Within the functionally annotated genes, no lysogenic lifecycle, antibiotic resistance- nor virulence-related genes could be retrieved, suggesting Arash can be safely used for biocontrol or therapy applications from a genomics perspective.

Since the vast majority of the genes remained unknown and a novel genus is proposed, a tandem mass spectrometry structural proteome analysis was performed (Table 2). Proteins with less than two unique identified peptides were considered as false positives and were not reported. Based on these criteria, 116 proteins could positively be identified as part of the mature phage particles. Of these, 27 were previously assigned a function based on similarity to known proteins. Eighty-eight were re-annotated as structural proteins, reducing the total percentage of ‘dark matter’ (i.e. unknown ORFs) in the genome from 80.2 to 48.0%.

The DNA-directed RNA polymerase (subunit beta) Gp206 is the protein detected the most, with 3516 total spectrum counts, followed by the structural proteins Gp4 and Gp229, the major capsid protein Gp226 and the beta’ subunit of the RNA polymerase Gp216 with respectively, 1952, 1345, 1333 and 1210 counts. Besides this beta and beta’ subunits, several enzymes are detected in the virus particles such as the cell wall hydrolase (Gp8), the RNA polymerase subunits sigma (Gp10), two ribonucleotide reductases (Gp27 and Gp61), two RNA ligases (Gp120 and Gp143) and a DNA helicase (Gp217) (Table 2). These enzymes might be directly needed at the start of infection such as the cell wall hydrolase for making a hole in the cell wall before injecting the phage DNA inside the host or e.g., the RNA polymerase for early transcription. Another possibility might be that these enzymes are expressed in large amounts during phage infection and are therefore still being present in the purified mature phage particles.

**Table 2:** Structural proteome of phage Arash. For each identified protein, the sequence coverage, the unique peptide count and the number of total detected spectra are reported. For proteins previously annotated as hypothetical, but now re-annotated as being structural, the functional annotation is italicized

Protein	Functional annotation	Coverage	# Unique Peptides	# Spectra
Gp004	*Structural protein*	87,88%	60	1952
Gp006	*Structural protein*	87,66%	16	280
Gp007	*Structural protein*	76,99%	7	202
Gp008	Cell wall hydrolase, structural protein	80,52%	44	777
Gp010	RNA polymerase sigma factor RpoS	100,00%	24	467
Gp011	*Structural protein*	100,00%	2	156
Gp012	*Structural protein*	77,38%	7	253
Gp013	*Structural protein*	82,95%	29	517
Gp014	*Structural protein*	60,44%	15	474
Gp015	*Structural protein*	76,22%	4	160
Gp016	*Structural protein*	100,00%	10	222
Gp017	*Structural protein*	64,67%	3	80
Gp019	Deoxynucleoside monophosphate kinase	100,00%	5	116
Gp021	*Structural protein*	89,13%	2	324
Gp023	Pyrophosphatase	53,14%	3	173
Gp024	*Structural protein*	97,60%	10	273
Gp026	*Structural protein*	100,00%	2	31
Gp027	Ribonucleotide reductase of class Ia (aerobic), alpha subunit	93,06%	11	663
Gp028	Phosphohydrolase	56,85%	2	56
Gp029	*Structural protein*	98,04%	3	90
Gp040	Pyrophosphohydrolase	99,64%	3	253
Gp042	*Structural protein*	73,38%	3	150
Gp046	*Structural protein*	100,00%	4	67
Gp047	*Structural protein*	89,03%	7	331
Gp049	*Structural protein*	100,00%	8	307
Gp050	*Structural protein*	73,08%	3	292
Gp051	*Structural protein*	100,00%	5	168
Gp052	*Structural protein*	99,16%	2	318
Gp057	*Structural protein*	100,00%	2	287
Gp058	Phosphoesterase	98,86%	3	76
Gp061	Ribonucleotide reductase of class Ia (aerobic), alpha subunit	100,00%	2	112
Gp065	*Structural protein*	76,28%	4	75
Gp067	*Structural protein*	77,48%	5	432
Gp070	*Structural protein*	100,00%	3	110
Gp071	*Structural protein*	95,40%	2	96
Gp082	*Structural protein*	87,50%	3	103
Gp089	*Structural protein*	100,00%	4	80
Gp092	HNH homing endonuclease	98,20%	2	193
Gp093	*Structural protein*	100,00%	3	115
Gp095	*Structural protein*	99,22%	5	406
Gp096	*Structural protein*	98,22%	15	816
Gp097	*Structural protein*	97,27%	24	1060
Gp100	*Structural protein*	98,84%	9	471
Gp103	*Structural protein*	100,00%	10	164
Gp113	*Structural protein*	90,58%	4	160
Gp117	*Structural protein*	84,29%	2	305
Gp118	*Structural protein*	88,76%	9	265
Gp120	RNA ligase	70,63%	3	300
Gp121	*Structural protein*	100,00%	3	209
Gp129	*Structural protein*	87,11%	4	246
Gp132	*Structural protein*	72,22%	6	121
Gp134	*Structural protein*	100,00%	2	91
Gp138	*Structural protein*	100,00%	9	180
Gp140	*Structural protein*	100,00%	13	334
Gp141	*Structural protein*	87,87%	26	836
Gp143	RNA ligase	100,00%	14	204
Gp149	*Structural protein*	100,00%	2	140
Gp151	*Structural protein*	100,00%	2	98
Gp156	*Structural protein*	100,00%	9	469
Gp157	Nicotinamide phosphoribosyltransferase	74,21%	3	423
Gp159	*Structural protein*	80,72%	5	195
Gp165	*Structural protein*	100,00%	2	254
Gp167	*Structural protein*	83,83%	2	179
Gp168	Uracil DNA glycosylase	98,66%	3	513
Gp169	*Structural protein*	55,03%	6	259
Gp173	*Structural protein*	100,00%	16	299
Gp177	DNA ligase	100,00%	2	495
Gp182	RNA exonuclease	90,24%	6	160
Gp183	DNA primase	89,74%	2	932
Gp185	*Structural protein*	100,00%	9	301
Gp186	*Structural protein*	100,00%	8	98
Gp187	*Structural protein*	100,00%	9	221
Gp188	*Structural protein*	100,00%	30	561
Gp190	Tail fiber protein	51,40%	4	92
Gp191	Tail protein	79,96%	58	832
Gp193	ATP-dependent DNA helicase	92,28%	2	217
Gp194	*Structural protein*	100,00%	9	159
Gp197	ATP-dependent DNA helicase	93,11%	2	517
Gp199	*Structural protein*	70,00%	9	245
Gp202	*Structural protein*	79,59%	6	125
Gp204	Portal protein	93,44%	32	877
Gp206	DNA-directed RNA polymerase subunit beta	100,00%	128	3516
Gp210	*Structural protein*	100,00%	7	111
Gp213	*Structural protein*	93,46%	7	143
Gp216	DNA-directed RNA polymerase subunit beta’	89,11%	40	1210
Gp217	DNA helicase	100,00%	38	1148
Gp218	*Structural protein*	89,11%	36	1149
Gp220	*Structural protein*	95,77%	6	289
Gp221	*Structural protein*	100,00%	8	355
Gp222	*Structural protein*	84,93%	16	365
Gp223	*Structural protein*	100,00%	9	283
Gp224	*Structural protein*	100,00%	8	222
Gp225	*Structural protein*	80,00%	2	203
Gp225.1	*Structural protein*	100,00%	14	582
Gp226	Major capsid protein	86,64%	30	1333
Gp227	Major tail protein	72,08%	17	406
Gp228	*Structural protein*	80,57%	12	117
Gp229	*Structural protein*	68,84%	79	1345
Gp230	*Structural protein*	60,49%	20	245
Gp231	*Structural protein*	97,09%	5	244
Gp232	*Structural protein*	100,00%	9	172
Gp233	*Structural protein*	100,00%	7	137
Gp234	*Structural protein*	87,16%	11	397
Gp235	*Structural protein*	100,00%	10	258
Gp236	*Structural protein*	80,59%	32	689
Gp237	*Structural protein*	89,57%	11	356
Gp238	*Structural protein*	90,26%	12	371
Gp239	PAAR-repeat central spike tip protein	100,00%	2	126
Gp242	*Structural protein*	100,00%	21	550
Gp243	*Structural protein*	100,00%	12	359
Gp244	*Structural protein*	80,50%	3	80
Gp246	*Structural protein*	100,00%	5	95
Gp247	*Structural protein*	77,17%	3	430
Gp248	*Structural protein*	61,41%	2	369
Gp249	*Structural protein*	100,00%	5	226
Gp251	*Structural protein*	76,25%	2	379

Generally phage Arash can be placed next to phages with a large genome or salmonella jumbo phages such as SPN3US, the collection of phages SPFM1 to SPFM22 and phage pSal-SNUABM-04. However Arash with an 180,819 bp genome is smaller than the mentioned phages. For instance, phage SPN3US, phages SPFM1 to SPFM22 and phage pSal-SNUABM possess gnomes sizes of 240,413, 233,195 to 242,624 and 239,626 bp, respectively. On the other hand Arash has a GC content of 53.02% while this for the mentioned phages was 48.54, 48.57 to 48.88 and 51.56%, respectively [54–56].

## Conclusion and perspectives

With the ever-increasing interest in the application of phages as antibacterial agents, the number of new phage genomes with little to no homology to any phages in the database will also keep on increasing. This creates unique opportunities to investigate the taxonomical similarity of bacteriophages based on their genome sequences. In this research, a novel phage from an unclassified, proposed genus ‘Arashvirus’ was isolated from the sewage of University Hospitals Leuven, Belgium and named phage Arash. Despite its narrow host range, Arash can be considered as a potential tool to deal with specific *S.* Typhimurium strains, both in the food industry and in human and animal infections, due to its many useful features, including its relatively fast adsorption, high burst size and the lack of antibiotic resistance, lysogenic and virulence factors in its genome.

### Electronic supplementary material

Below is the link to the electronic supplementary material.

Supplementary Material 1

Supplementary Material 2

## Data Availability

The *S.* Typhimurium phage genome sequence was deposited in NCBI. The accession number is OQ632216.
