# Supplementary material for: Isolation and molecular characterization of the Salmonella Typhimurium orphan phage Arash
Source: BMC Microbiol. 2023 Oct 19;23:297. doi: 10.1186/s12866-023-03056-9 (PMC10585845; doi:10.1186/s12866-023-03056-9)
Supplement: Supplementary file 1 — Supplementary Material 1 [file 12866_2023_3056_MOESM1_ESM.docx]

**Supplementary Figures**

**Figure S1:** plaques of Phage Arash on bacterial lawn of Salmonella Typhimurium ATCC 14028


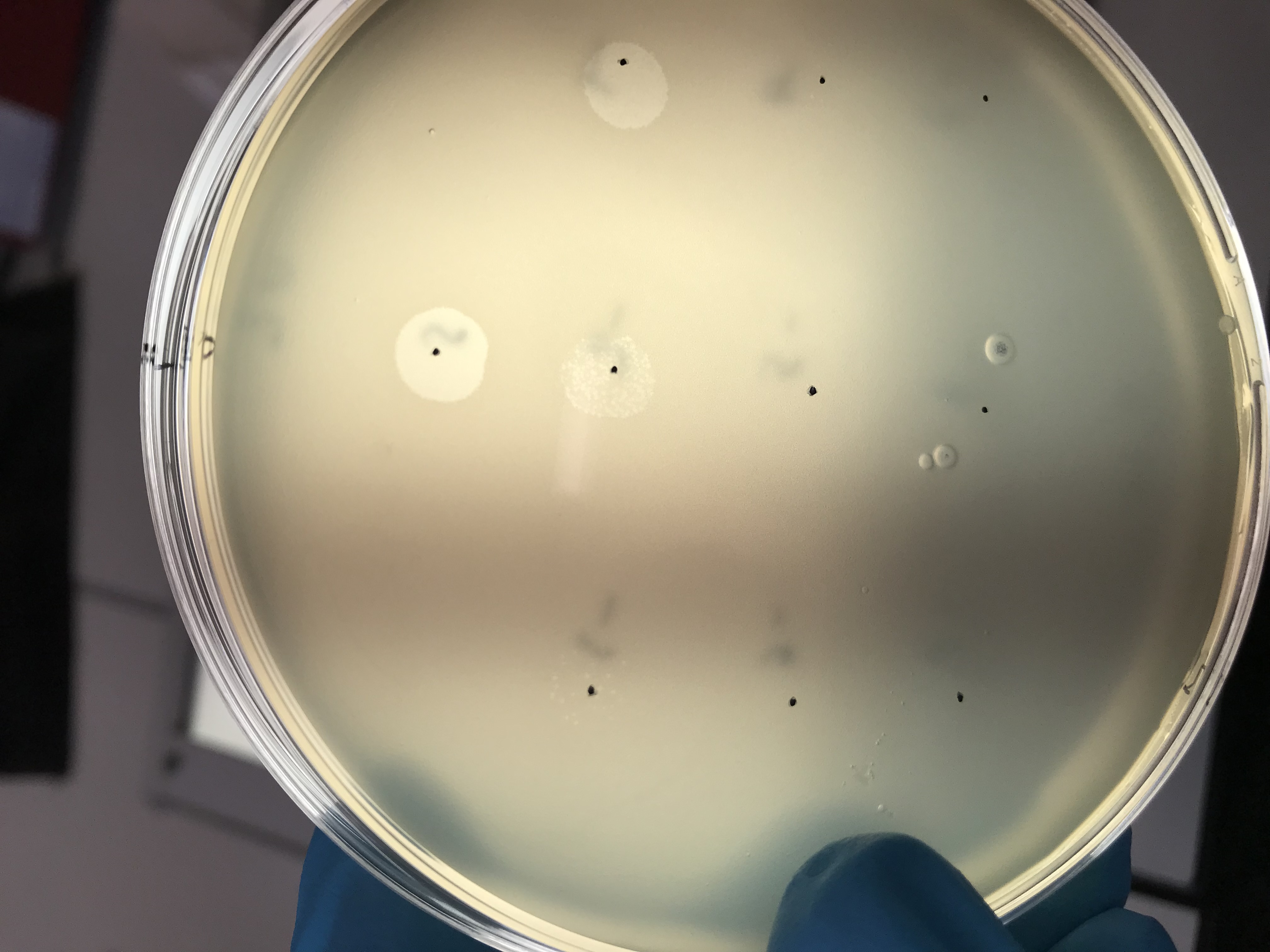

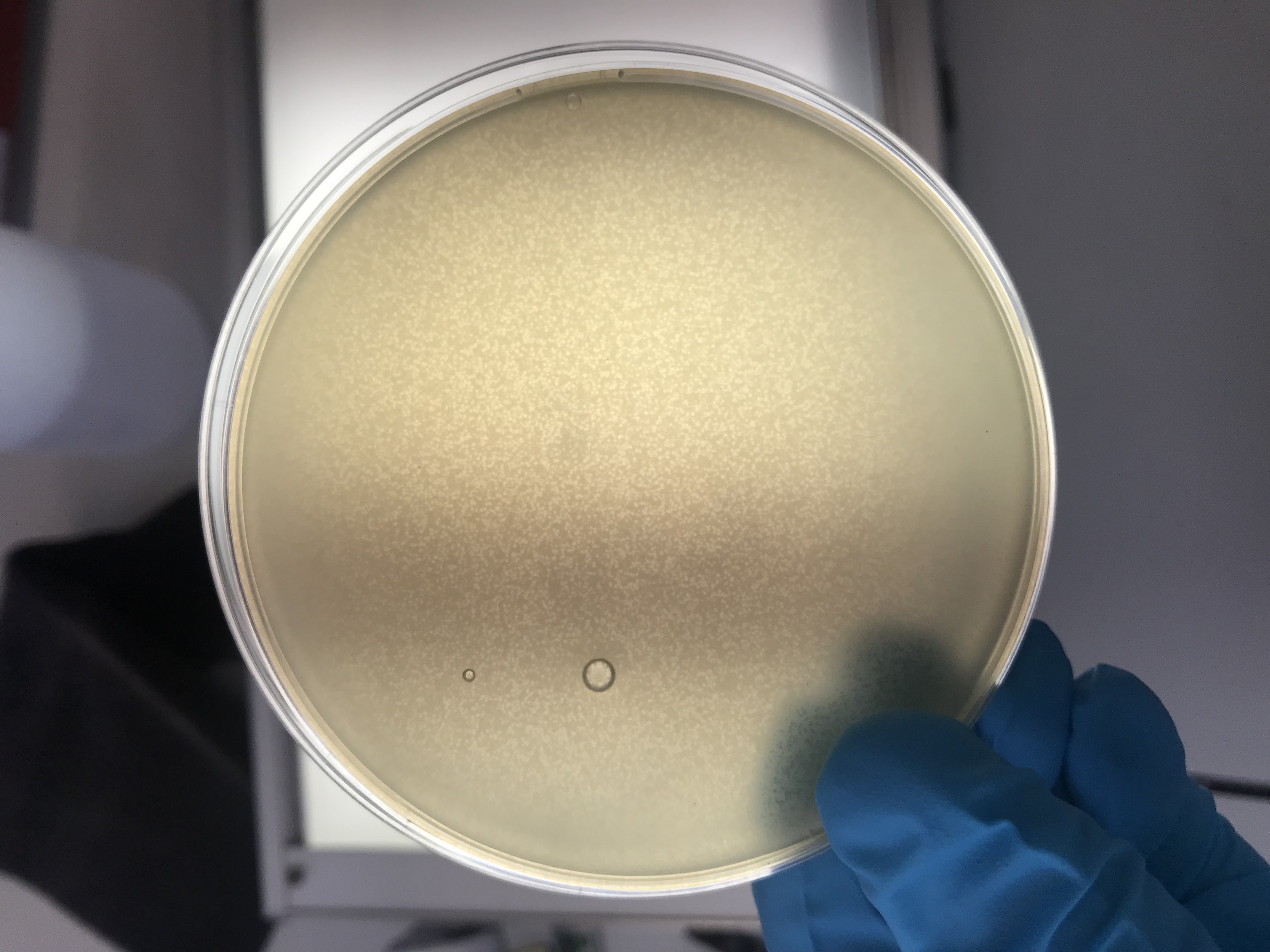

**Figure S2.** VIRIDIC (Virus Intergenomic Distance Calculator) heatmap comparing the phages most related to Arash. The number in the matrix (right side) shows the intergenomic similarity between each pair of phages as a percentage of sequence identity. Phages sharing more than 95% and 70% identity are members of the same species and genus, respectively.
